# MiR‐520b promotes the progression of non‐small cell lung cancer through activating Hedgehog pathway

**DOI:** 10.1111/jcmm.13909

**Published:** 2018-11-08

**Authors:** Xiaoming Liu, Jichun Liu, Xuekang Zhang, Yuben Tong, Xin Gan

**Affiliations:** ^1^ Department of Cardiothoracic Surgery The First Affiliated Hospital of Nanchang University Nanchang Jiangxi China; ^2^ Department of Anesthesiology The First Affiliated Hospital of Nanchang University Nanchang Jiangxi China; ^3^ Department of Respiration The First Affiliated Hospital of Nanchang University Nanchang Jiangxi China

**Keywords:** Hh pathway, miR‐520b, NSCLC, SPOP

## Abstract

Although the non‐small cell lung cancer (NSCLC) is one of the most malignant tumours worldwide, the mechanisms controlling NSCLC tumourigenesis remain unclear. Here, we find that the expression of miR‐520b is up‐regulated in NSCLC samples. Further studies have revealed that miR‐520b promotes the proliferation and metastasis of NSCLC cells. In addition, miR‐520b activates Hedgehog (Hh) pathway. Inhibitor of Hh pathway could relieve the oncogenic effect of miR‐520b upon NSCLC cells. Mechanistically, we demonstrate that miR‐520b directly targets *SPOP* 3′‐UTR and decreases *SPOP* expression, culminating in GLI2/3 stabilization and Hh pathway hyperactivation. Collectively, our findings unveil that miR‐520b promotes NSCLC tumourigenesis through SPOP‐GLI2/3 axis and provide miR‐520b as a potential diagnostic biomarker and therapeutic target for NSCLC.

## 1 INTRODUCTION

Lung cancer is the most frequent cause of cancer‐related death in men and the second most frequent in women after breast cancer. Many risk factors are strong correlated with lung cancer tumourigenesis, including smoking and air pollution.^1^, ^2^ Despite significant advancements have been made in surgeries, radiotherapies and chemotherapies in the past decades, the survival rate of lung cancer is still low.^3^ Lung cancers comprise two types, small cell lung cancer (SCLC) and non‐small cell lung cancer (NSCLC).^4^ NSCLC contributes to over 80% of lung cancers. In past years, although many genes, such as *P53* and *EGFR*, have been reported to regulate NSCLC development, most of NSCLS patients are diagnosed at the late stage and die without successful treatment. Thus, studies on the mechanisms of NSCLC tumourigenesis are critical for both early prognosis and the development of novel therapeutic approaches for NSCLC.

In NSCLC tumourigenesis, multiple pathways are deregulated due to genetic or epigenetic mutations. Constitutive activation of Shh pathway is a common cause of NSCLC.^5^ Lots of mutations on Hh signalling components have identified in NSCLC samples.^6^, ^7^ Besides, several Hh pathway inhibitors, including Cyclopamine and Forskolin, serve as attractive anti‐cancer agents for NSCLC treatment.^8^, ^9^, ^10^ The Hh pathway is first identified through a large gene screening that is required for embryonic patterning in *Drosophila*.^11^ Subsequent studies show that the Hh pathway is highly conserved from *Drosophila* to mammals.^12^ In the absence of Hh ligand, the transcriptional factor GLI2/3 is detained in the cytoplasm to degrade by β‐TrCP E3 ligase. In the presence of Hh ligand, GLI2/3 translocates into the nucleus to turn on the expression of target genes, including *PTCH1* and *BCL2*.^13^ On the other hand, the nuclear GLI2/3 is ubiquitinated and destabilized by another E3 ligase SPOP,^14^ ensuring that the proper Hh pathway output. For this respect, SPOP plays an anti‐tumour role in Hh‐related NSCLC.

Micro RNAs (miRNAs) are small (about 19‐25 bp), noncoding RNAs that silence gene expression through repression of mRNA stability or translation. Recent studies have demonstrated that many miRNAs show aberrant expression in tumour tissues, indicating that miRNAs are possibly involved in tumourigenesis. On the other hand, miRNAs also provide putative biomarkers for tumour diagnoses.^15^ Increasing findings show that several miRNAs plays important roles in NSCLC tumourigenesis. MiR‐21 promotes NSCLC cell proliferation and invasion through inhibiting the expression of *PTEN*, a well‐known tumour suppressor.^16^ In contrast, miR‐1253 suppresses NSCLC progression via blocking *WNT5A* expression.^17^ Therefore, miRNAs likely play dual roles in NSCLC tumourigenesis through silencing distinct targets. Although human genome encodes more than 400 kinds of miRNAs, the functions of many miRNAs are still unknown. It will be fruitful to investigate the roles of miRNAs in NSCLC progression.

In this study, we found that the expression of miR‐520b was apparently up‐regulated in NSCLC samples, and miR‐520b levels were positively correlated with Hh pathway activities. In NSCLC cells, treated with miR‐520b mimic promoted cell proliferation and migration, while miR‐520b inhibitor treatment showed an opposite effect. Via bioinformatics and biochemical analyses, we revealed that miR‐520b suppressed *SPOP* expression through direct targeting its 3′‐UTR region. In addition, SPOP decreased in NSCLC samples and negative correlated with patient survival. Knockout of *SPOP* promoted NSCLC cell proliferation and metastasis, which could not be attenuated by miR‐520b, suggesting that miR‐520b promotes NSCLC progression through SPOP. Finally, we demonstrated that SPOP inhibited NSCLC tumourigenesis through ubiquitinating and destabilizing GLI2/3. Taken together, our findings show that miR‐520b promotes NSCLC tumourigenesis via SPOP‐GLI2/3 axis, thus providing miR‐520b as a potential diagnostic biomarker and therapeutic target for NSCLC.

## 2 MATERIALS AND METHODS

### 2.1 NSCLC cell lines and patient samples

Human NSCLC cell lines (A549 and H1299) were purchased from the ATCC and cultured in Dulbecco's modified Eagle's medium (Gibco, Gaithersburg, MD, USA) containing 10% FBS and 1% penicillin/streptomycin (Sangon Biotech, Shanghai, China). MiR‐520b mimic (MC11115) and inhibitor (MH11115) were purchase from Thermo and added into the cells at final concentration of 20 nmol/L. Fresh‐frozen NSCLC samples and their paired normal samples were obtained from patients who were undergoing surgical resection at the First Affiliated Hospital of Nanchang University (Nanchang, China). None of the patients had received any radiochemotherapy. All the samples were divided into two parts for RNA extraction and western blot, respectively. The use of human samples was approved by Clinical Research Ethics Committee of Nanchang University, China.

### 2.2 In vitro tumorigenicity assay

Cell proliferation was assessed using MTT assay. After distinct treatments, log‐phase cells were seeded into 96‐well plates. After additional 48 hours, 10 mL MTT (5 mg/mL) was added into each well, followed by incubation for 4 hours before discarding the supernatants. Washing the cells with PBS for three times and adding 100 mL DMSO in each well to dissolve crystals for 10 minutes. The absorbance on 490 nm was measured using microplate reader.

The cell invasion assay was performed with Biocoat Matrigel Invasion Chambers. After indicated treatments, equivalent cells were seeded on top of a thick layer of Matrigel in transwell inserts and cultured for 24 hours. Invasive cells were washed with PBS, fixed with 70% ethanol for 15 minutes and stained with 2% crystal violet. The invasive cells were counted under a microscope.

Cell migration was tested using wound healing assay. After indicated treatments, equivalent cells were seeded into 6‐well plates with 1% FBS. One yellow pipette tip was used to make a straight scratch. The width of wound was measured at 48 hours and normalized with starting time‐point.

### 2.3 RNA extraction and quantitative real‐time PCR (Q‐PCR)

Total RNA from patient samples and cultured cells was extracted using TRIzol reagent. High‐capacity cDNA reverse transcription kit was used for cDNA synthesis. Q‐PCR was conducted on a CFX96™ with SYBR Green Q‐PCR reagents. The 2‐ΔΔCt method was used for relative quantification. The primer pairs used were as follows: *GLI1*, 5′‐GGGTGCCGGAAGTCATACTC‐3′ (forward) and 5′‐GCTAGGA‐TCTGTATAGCGTTTGG‐3′ (reverse); *BCL2*, 5′‐CTCAGCAGGTATCACATGG‐GG‐3′ (forward) and 5′‐CCAAGGTCTTGCGTACAAATTCC‐3′ (reverse); *ACTIN*, 5′‐GATCATTGCTCCTCCTGAGC‐3′ (forward) and 5′‐ACTCCTGCTTGCTGAT‐CCAC‐3′ (reverse).

For miRNA expression detection, Taqman miRNA assays were employed to quantify the expression of mature miR‐520b. The relative expression level of miR‐520b was normalized to RNU6B.

### 2.4 Constructs, Cas9 and RNAi

To generate Fg‐SPOP, Myc‐GLI2, Myc‐GLI3 and HA‐GLI3 constructs, we amplified the corresponding cDNA using Primer STAR DNA polymerase (TAKARA, Kusatsu,Shiga, Japan) and then cloned them into CMV‐Fg, pcDNA3.1‐Myc or pcDNA3.1‐HA vectors respectively. SPOP mutant plasmids were generated using PCR‐based site‐directed mutagenesis at the background of CMV‐Fg‐SPOP.

To knock out the endogenous *SPOP*, we used CRISPR/Cas9 tool. The sgRNA targeting SPOP was CCTCCGGCAGAAATGTCGAGTGG. It was annealed to the complementary oligo and cloned into pGL3‐U6‐sgRNA‐PGK‐puromycin vector (Addgene, Cambridge, MA, USA). A549 cells were cotransfected with this plasmid and pST‐NLS‐Cas9 plasmid (Addgene). 48 hours after transfection, the cells were treated by puromycin (0.02 mg/mL, Invivogen, San Diego, USA) and blasticidin (0.75 mg/mL, Invivogen) for additional 48 hours. After cells form colonies, pick the small colonies into 96‐well plates. Genomic DNA from the cells is amplified by PCR. Putative mutants were further validated by sequencing.

To silence *SPOP*,* GLI2* or *GLI3* in NSCLC cells, small interfering RNAs (siRNAs) were transfected at a final concentration of 200 nmol/L via Lipofectamine™ RNAiMAX transfection reagent according to the protocols (Invitrogen, Carlsbad, CA, USA). The siRNAs sequences were shown as follows: *CTR*‐siRNA, 5′‐CAAACACUUCCUUGGAAUGdTdT‐3′; *SPOP*‐siRNA‐1, 5′‐CUCACCGGGGCAUCGACUCdTdT‐3′; *SPOP*‐siRNA‐2, 5′‐G‐GUUUCGAUACCUCUCAGUdTdT‐3′; *GLI2*‐siRNA, 5′‐GUUCCUCACGGCGU‐ CGUAGdTdT‐3′ and *GLI3*‐siRNA, 5′‐UGGAAGUUGUAGCUCACUGdTdT‐3′.

### 2.5 Transfection and western blot

Cells were transfected using lipofectamine 2000 according to the manufacturer's instructions. Forty‐eight hours after transfection, cells were harvested for immunoprecipitation and western blot analysis with standard protocols. To examine the ubiquitination levels of GLI2 and GLI3, A549 cells were transfected with Myc‐GLI2, HA‐GLI3 and different SPOP mutants. Before cell harvesting, the cells were treated by MG132 (50 mmol/L/mL) for 4 hours to prevent protein destabilization. Cells were first lysed by 100 mL denaturing buffer (1% SDS, 50 mM Tris, pH 7.5, 0.5 mmol/L EDTA and 1 mmol/L DTT) and incubated at 100°C for 5 minutes. The lysates were diluted with 900 mL lysis buffer and subjected to immunoprecipitation and western blot. The antibodies used for western blot analyses were as follows: mouse anti‐Fg (Sigma, Darmstadt, Germany); mouse anti‐ACTIN (Genscript, Corporation, Piscataway, NJ, USA); rabbit anti‐GLI1 (ABclonal, Woburn, MA, USA); rabbit anti‐PTCH1 (ABclonal); rabbit anti‐BCL2 (ABclonal); rabbit anti‐HHIP (ABclonal); rabbit anti‐AXIN2 (ABclonal); rabbit anti‐c‐Myc (ABclonal); rabbit anti‐CTGF (ABclonal); rabbit anti‐AREG (ABclonal); rabbit anti‐SPOP (ABclonal); mouse anti‐Myc (Santa Cruz Biotechnology, Santa Cruz, CA, USA); mouse anti‐HA (Santa Cruz); mouse anti‐Ub (Santa Cruz); goat antimouse HRP (Abmax) and goat anti‐rabbit HRP (Abmax, Beijing, China).

### 2.6 Luciferase assays

The putative miR‐520b binding site in 3′‐UTR of *SPOP* was subcloned into pGL3‐Basic‐Luc vector (Promega, Woods Hollow Road, USA). Meanwhile, the corresponding mutant construct was generated by mutation of the complementary sequence of miR‐520b seed region (AGCACTTA to TCGTGAAT). The firefly luciferase construct was cotransfected with Renilla luciferase plasmid into A549 cells. Dual Luciferase Reporter Assay System was employed to check the luciferase activity after 24 hours according to the manufacturer's instruction. All luciferase activity data are presented as means ± SD from at least three independent experiments.

### 2.7 Statistical analysis

All statistical analysis was performed by SPSS software. The reported data are representative of at least three independent experiments. A two‐tailed *P* value of less than 0.05 was considered statistically significant and the *P* < 0.001 was considered highly significant. In this study, exact *P* values were not shown, statistical significance was as follows: *P *>* *0.05 (NS, no significance), *P *<* *0.05 (*), *P *<* *0.01 (**) and *P *<* *0.001 (***).

## 3 RESULTS

### 3.1 MiR‐520b is up‐regulated and plays a oncogenic role in NSCLC

**Figure 1:**
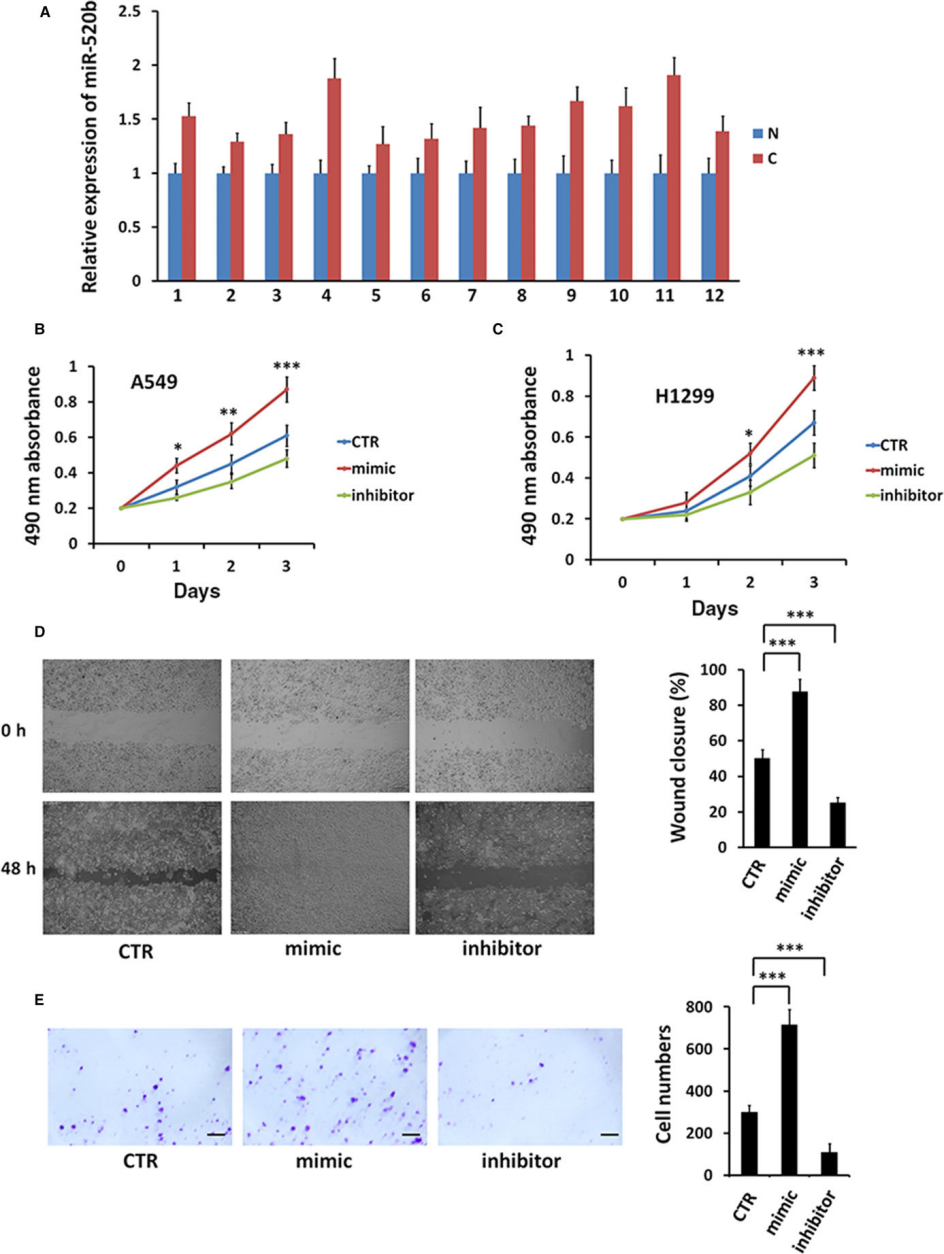
miR‐520b is up‐regulated in NSCLC samples and exerts an oncogenic role in NSCLC cells. (A) Q‐PCR analysis of miR‐520b expression in 12 pairs of NSCLC samples and paratumour normal tissues (N, paratumour normal samples; C, cancer samples). (B) 3‐day MTT proliferation results of A549 cells with miR‐520b mimic or miR‐520b inhibitor treatment. (C) MTT assays of H1299 cells treated with miR‐520b mimic or miR‐520b inhibitor. (D) Wound healing assays of A549 cells treated with miR‐520b mimic or miR‐520b inhibitor. Quantification of wound closure at indicated time‐points was shown on the right. (E) Transwell assay of A549 cells treated with miR‐520b mimic or miR‐520b inhibitor. Numbers of invasive cells were shown on the right. Scale bar, 20 μm. All values are mean ± SD (n = 3, *P <* *0.05,**P < 0.01 and ***P < 0.001)

Although it is known miR‐520b plays roles in several types of cancer, its function in NSCLC is still unknown. We examined the expression of miR‐520b in NSCLC patients using Q‐PCR and found that miR‐520 was uniformly increased in 12 NSLCL samples compared with the paratumour samples (Figure 1A). To further investigate the function of miR‐520b, we treated the NSCLC cells with miR‐520b mimic or miR‐520b inhibitor. Compared with control, miR‐520b mimic promoted, while miR‐520b inhibitor suppressed cell proliferation of A549 and H1299 cells (Figure 1B, C). In addition, wound healing assay showed that miR‐520b mimic quickened the wound healing, but miR‐520b inhibitor exerted an opposite effect (Figure 1D). Consistently, transwell results revealed that miR‐520b mimic increased, whereas miR‐520b inhibitor decreased H1299 cell invasion (Figure 1E). Taken together, our results reveal that miR‐520b is up‐regulated in NSCLC samples and miR‐520b exerts oncogenic function in NSCLC cells.

### 3.2 MiR‐520b activates Hh pathway

**Figure 2:**
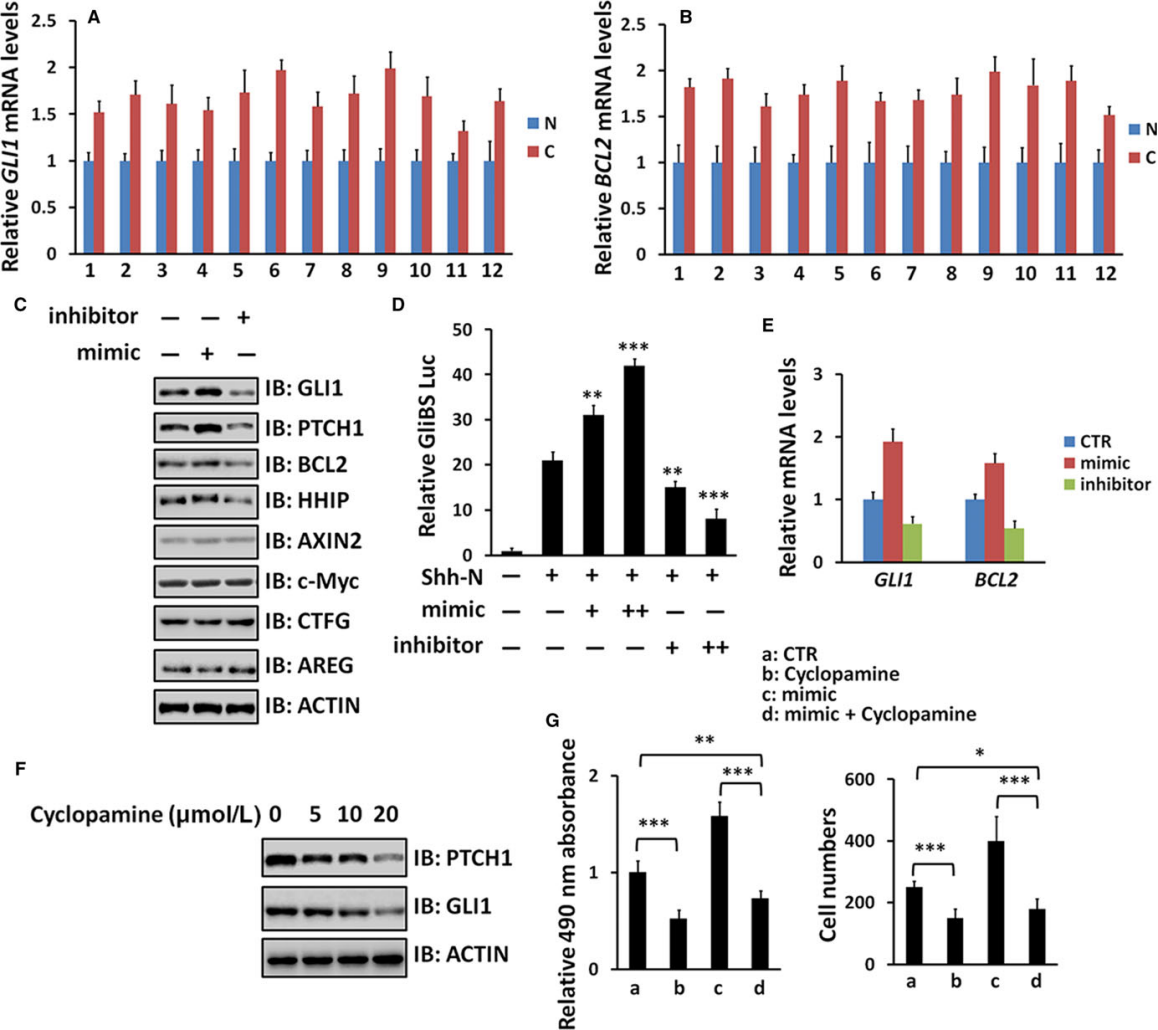
miR‐520b activates Hh pathway in NSCLC cells. (A, B) Relative mRNA levels of *GLI1* and *BCL2* from NSCLC samples and matched normal samples were examined by Q‐PCR. (C) WB results of A549 cells with miR‐520b mimic or miR‐520b inhibitor treatment. (D) GliBS‐luciferase reporter assay in A549 cells under indicated treatment. GliBS luciferase activities were normalized to Renilla luciferase activities. (E) Q‐PCR assay to test *GLI1* and *BCL2* with miR‐520b mimic or miR‐520b inhibitor treatment in A549 cells. (F) WB results of A549 cells treated by Cyclopamine at indicated concentrations. (G) MTT and transwell assays of A549 cells under indicated treatment. All values are mean ± SD (n = 3, *P < 0.05,**P < 0.01 and ***P < 0.001)

During NSCLC tumourigenesis, multiple oncogenic pathways are involved in, including Hh, Wnt and Hippo.^18^, ^19^, ^20^ We next wanted to test whether miR‐520b turns on these pathways. We found that the expressions of Hh pathway targets (*GLI1* and *BCL2*) were apparently increased in NSCLC specimens (Figure 2A, B). Furthermore, miR‐520b mimic treatment indeed increased Hh target gene expression (*GLI1*,* PTCH1*,* BCL2* and *HHIP*), but with no any detectable effect on Wnt pathway (*AXIN2* and *c‐Myc*) and Hippo pathway (*CTGF* and *AREG*) (Figure 2C). In contrast, the inhibitor attenuated Hh pathway, not Wnt and Hippo pathways (Figure 2C). Furthermore, we found that the mimic elevated Gli‐Luciferase activity, while the inhibitor played an opposite role (Figure 2D). Q‐PCR in A549 cells also confirmed these results (Figure 2E). These data suggest that miR‐520b possibly specifically activates Hh pathway in NSCLC cells.

To test whether miR‐520b exerts the oncogenic effect through Hh signalling, a well‐known Hh antagonist cyclopamine was employed to block Hh pathway. Treated A549 cells with distinct concentrations of cyclopamine indeed decreased the expression of *PTCH1* and *GLI1* (Figure 2F), ensuring the efficiency of this compound. The up‐regulated proliferation and invasion caused by miR‐520b mimic was neutralized by cyclopamine (Figure 2G), indicating that miR‐520b promotes NSCLC cell proliferation and invasion through Hh signalling.

### 3.3 SPOP is a target of miR‐520b

**Figure 3:**
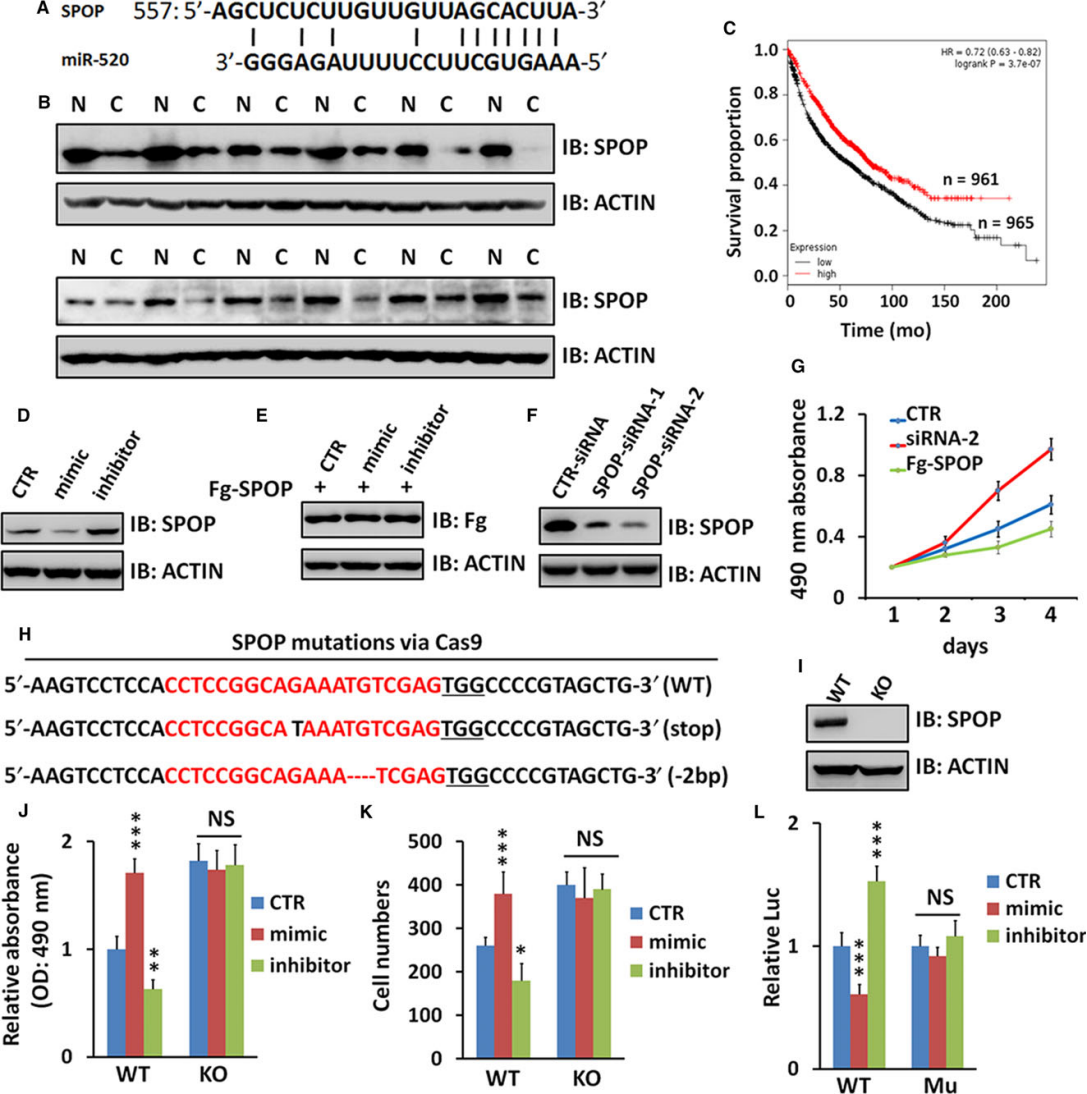
miR‐520b directly targets *SPOP* in NSCLC cells. (A) A putative miR‐520b binding site in the 3′‐UTR of *SPOP*. (B) WB analyses of SPOP protein levels in 12 pairs of NSCLC samples and paratumour normal samples. (C) Low miR‐520b expression was associated with a poor disease free survival in TCGA cohort (*P* < 0.0001). (D) WB assay of A549 cells treated by miR‐520b mimic or miR‐520b inhibitor. (E) Neither miR‐520b mimic nor miR‐520b inhibitor affects exogenous SPOP expression in A549 cells. (F) WB assay showed SPOP protein level with *SPOP*‐siRNA treatment. (G) MTT assays of A549 cells transfected with indicated siRNA or plasmid. (H) Alignment of Sanger sequencing results of PCR amplicons encompassing the target site, SgRNAs targets are highlighted in red and the PAM sequence is underlined. (I) WB analysis of SPOP expression of WT or KO A549 cells. (J) MTT analyses of WT or *SPOP* KO A549 cells treated with miR‐520b mimic or miR‐520b inhibitor. (K) Transwell analyses of WT or *SPOP* KO A549 cells treated with miR‐520b mimic or miR‐520b inhibitor. (L) miR‐520b only suppressed the relative luciferase activity in the construct, which contains the wild‐type sequence of the binding site in the 3′‐UTR of *SPOP*. WT, luciferase construct containing wild‐type binding site in *SPOP* 3′‐UTR; Mu, mutated nucleotides were introduced to the complementary seed sequence. All values are mean ± SD (n = 3, *P < 0.05, ***P < 0.001 and NS, no significance)

To investigate the target of miR‐520b, we employed bioinformatic analysis (www.targesan.org) and found *SPOP* was a putative target (Figure 3A). We demonstrated that the expression of SPOP was decreased in NSCLC samples (Figure 3B), showing negative correlation with miR‐520b. Consistently, data from TCGA (http://cancergenome.nih.gov/) revealed that patients with low SPOP expression (n = 965) had a poorer survival compared with those with high miR‐520b expression (n = 961, *P *<* *0.0001) (Figure 3C). Furthermore, we found that miR‐520b mimic decreased, while miR‐520b inhibitor increased SPOP protein level in A549 cells (Figure 3D). On the other hand, neither mimic nor inhibitor showed any effect on exogenous SPOP level (Figure 3E), suggesting that miR‐520b decreases SPOP through targeting *SPOP* 3′‐UTR region.

To examine the anti‐tumour role of SPOP on NSCLC, we silenced endogenous SPOP via siRNA and found that knockdown of SPOP promoted A549 cell proliferation, whereas overexpression of SPOP inhibited cell proliferation (Figure 3G). In addition, CRISPR/Cas9 technique was used to knock out *SPOP* in A549 cells (Figure 3H). We picked up 10 cell lines and found a stop codon was induced in a cell line (Figure 3H). Western blot analysis showed complete depletion of SPOP protein in the (stop) cell line. Therefore, we chose this cell line (stop) for subsequent experiments. Knockout of *SPOP* indeed promoted cell proliferation (Figure 3J) and invasion (Figure 3K), and this oncogenic effect could not be relieved by miR‐520b mimic or miR‐520b inhibitor (Figure 3J, K). Furthermore, A549 cells with *SPOP* knockout failed to be modulated by miR‐520b (Figure 3J, K), suggesting that SPOP is essential for miR‐520 regulating NSCLC cell proliferation and invasion. To test whether SPOP is the direct target of miR‐520b, luciferase reporter assays were performed. The fragment of the *SPOP* 3′‐UTR containing the predicted or mutant miR‐520b site was cloned into the pGL3‐Basic‐Luc vector. We found that miR‐520b exerted inhibitory effects on the luciferase activity in the construct which contains the wild‐type binding site, whereas no suppressive effects on the binding site mutant construct (Figure 3L).

### 3.4 SPOP suppresses Hh pathway through ubiquitinating GLI2 and GLI3

**Figure 4:**
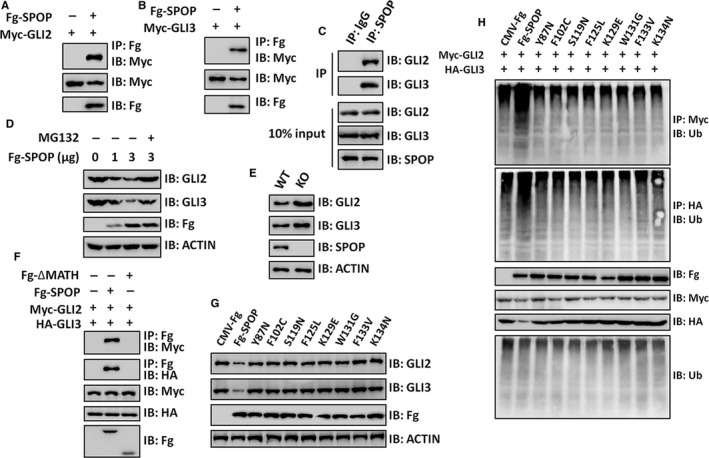
SPOP promotes GLI2/3 ubiquitination and degradation. (A, B) Fg‐SPOP could pull down Myc‐GLI2 (A) and Myc‐GLI3 (B) in A549 cells. (C) Endogenous SPOP interacted with endogenous GLI2 and GLI3 in A549 cells. (D) SPOP promoted GLI2/3 degradation in A549 cells, which was abolished by MG132 treatment. (E) WB analysis of WT and *SPOP* KO A549 cells. (F) SPOP bound GLI2/3 through its N‐terminal MATH domain. (G) WB assay of A549 cells transfected with WT SPOP or different SPOP mutants. (H) Ubiquitination assay of A549 cells transfected with indicated plasmids

The previous studies have demonstrated that Rdx (SPOP homologue) binds and ubiquitinates Ci (GLI2/3 homologue) to inhibit Hh signalling activity in *Drosophila*.^14^ We tried to test whether SPOP destabilizes GLI2/3 to negatively regulate Hh pathway in NSCLC cells. In A549 cells, SPOP could bind GLI2/3 (Figure 4A, B), and promoted proteasome‐mediated GLI2/3 degradation in a dose‐dependent manner (Figure 4C). Moreover, GLI2/3 showed increased expression in SPOP knockout cells compared with wild‐type cells (Figure 4D). SPOP protein comprises two functional domains: the N‐terminal MATH domain and the C‐terminal BTB domain. Loss of MATH domain deprived SPOP association with GLI2/3 (Figure 4E), indicating that SPOP interacts with GLI2/3 via its N‐terminal MATH domain.

Previous GWAS screening has shown that *SPOP* is mutated in several human cancers, including prostate cancer and gastric cancer.^21^, ^22^ Interestingly, most *SPOP* mutations localize in the MATH domain, which presumably impair its ability to bind substrates. To explore whether the cancer‐derived mutations affect SPOP interaction with GLI2/3, we carried out co‐IP assays and found that only wild‐type SPOP interacted with GLI2/3 (Figure 4F). Given that SPOP acts as an E3 ligase, we next wanted to examine the ubiquitination level of GLI2/3. Consistently, SPOP indeed elevated the ubiquitination of GLI2/3, whereas these SPOP mutants failed to promote GLI2/3 ubiquitination (Figure 4G). Collectively, these results show that SPOP inhibits Hh pathway through destabilizing the transcriptional factor GLI2/3, which is abolished in cancers due to SPOP mutations.

### 3.5 MiR‐520b regulates NSCLC through SPOP‐GLI2/3 axis

**Figure 5:**
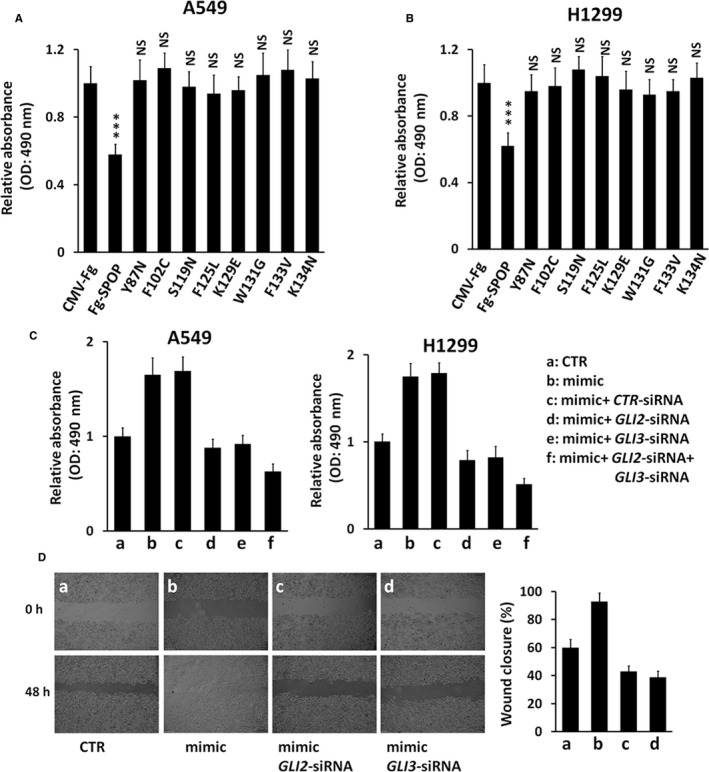
miR‐520b exerts oncogenic effect on NSCLC cells through GLI2/3. (A, B) MTT assays of A549 cells (A) and H1299 cells (B) transfected with indicated constructs. (C) MTT assays of A549 cells (left) and H1299 cells (right) under indicated treatment. (D) Wound healing assays of A549 cells under indicated treatment. Quantification of wound closure at indicated time‐points was shown on the right. All values are mean ± SD (n = 3, ***P < 0.001 and NS, no significance)

The above results have clearly demonstrated that mutations on MATH domain destroy SPOP binding to GLI2/3. Consistently, wild‐type SPOP attenuated the proliferation of A549 and H1299 cells, whereas SPOP mutants did not show any affects (Figure 5A, B), indicating that SPOP regulates NSCLC cell proliferation possibly through binding and ubiquitinating GLI2/3. We next wanted to explore whether miR‐520b plays oncogenic roles via GLI2/3. The up‐regulated cell proliferation caused by miR‐520b mimic was effectively restored by silencing GLI2 or GLI3 (Figure 5C), suggesting that miR‐520b sits upstream of GLI2/3 to promote NSCLC cell proliferation. Furthermore, wound healing assays also showed that miR‐520b promoted NSCLC cell migration through GLI2/3.

## 4 DISCUSSION

NSCLC is one of the most malignant cancers and leads to many deaths every year. It is a difficult task to diagnose the NSCLC at early stage owing to lack of apparent symptoms. Thus, it is urgent to identify the bio‐marker for NSCLC early diagnosis. In this study, we found that miR‐520b was up‐regulated in NSCLC specimens and positively correlated with NSCLC cell proliferation, migration and invasion. Furthermore, we demonstrated that miR‐520b could activate Hh signalling in NSCLC cells. Through bioinformatic and biochemical analyses, we revealed that *SPOP* was a target of miR‐520b. SPOP bound GLI2/3 via its N‐terminal MATH domain, resulting in GLI2/3 proteasome‐mediated degradation and Hh pathway inactivation. Finally, we found that knockdown of *GLI2/3* effectively neutralized the effects of miR‐520b on NSCLC cell proliferation and migration. Taken together, our findings illustrated that miR‐520b exerted oncogenic effect in NSCLC through SPOP‐GLI2/3 signalling axis. This study provides miR‐520b as a putative bio‐marker for NSCLC diagnosis and therapeutic target for NSCLC clinic intervention.

As a matter of fact, accumulating studies show that miR‐520b plays important roles in tumourigenesis. In oestrogen receptor negative breast cancer, miR‐520b functions as a tumour suppressor by targeting NF‐κB and TGF‐β pathways.^23^ Besides, it is also reported that miR‐520b suppresses cell migration and invasion through inhibiting CD44 in head‐neck cancer.^24^ Based on previous data and our present study, miR‐520b likely plays oncogenic or anti‐tumour roles in different cancers through targeting distinct genes. It will be fruitful to explore the roles of miR‐520b in other cancers.

The Hh pathway is a central regulator of development and tumourigenesis.^25^ During embryogenesis, Hh pathway is indispensable for the development of multiple tissues, including brain, limbs and lung.^26^, ^27^, ^28^ In adult, Hh signalling keeps basal activity to regulate injury‐induce regeneration and stem cell maintenance. Its hyperactivation will lead to tumourigenesis.^29^ Although Hh pathway is a key inducer for cancers, its inhibitors are still difficult for clinic application since it is involved in multiple physiological and pathological processes. In this study, we found that miR‐520b promoted NSCLC progression by activating Hh pathway. Thus, we possibly choose miR‐520b as a therapeutic target for Hh‐related NSCLC patients.

**Figure 6:**
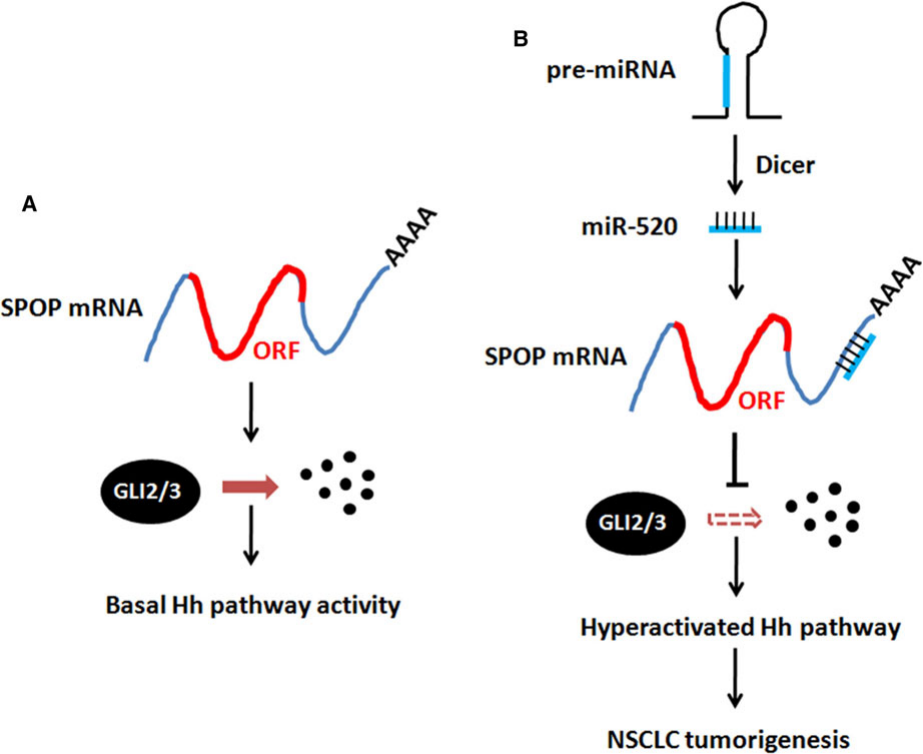
The model of miR‐520b regulating NSCLC tumourigenesis. (A) In normal lung cells, SPOP ubiquitinates GLI2/3, resulting in GLI2/3 degradation and Hh pathway suppression. Under this condition, Hh pathway activity keeps at the basal level. (B) In NSCLC cells, miR‐520b is up‐regulated and targets *SPO*P mRNA, removing SPOP‐mediated GLI2/3 destabilization. In turn, Hh pathway is hyperactivated

SPOP is a well‐known E3 ligase, which promotes substrate ubiquitination and degradation. Increasing substrates of SPOP are reported, such as PD‐L1, DAXX, c‐Myc and PTEN.^30^, ^31^, ^32^, ^33^ More attention has been paid to identify novel substrates of SPOP, but the regulation of SPOP is still unknown. In this study, we found that miR‐520b was a negative regulator of SPOP through silencing *SPOP* expression. In normal lung cells, the Hh pathway keeps low activity through SPOP‐mediated GLI2/3 degradation (Figure 6). In NSCLC cells, the expression of miR‐520b is up‐regulated. MiR‐520b targets *SPOP* to block SPOP‐mediated GLI2/3 destabilization, culminating in Hh signalling hyperactivation and NSCLC tumourigenesis (Figure 6). In this model, miR‐520b plays a key role during NSCLC tumourigenesis.

## CONFLICT OF INTERESTS

The authors declare that they have no competing interests.

## AUTHOR CONTRIBUTION

XL and JL performed the majority of experiments, data analysis. XZ constructed many plasmids used in this study. YT collected the patient samples and carried out some Q‐PCR experiments. XG supervised the study and wrote the manuscript.

## References

[1] Lemjabbar‐Alaoui H , Dasari V , Sidhu SS , et al. Wnt and Hedgehog are critical mediators of cigarette smoke‐induced lung cancer. PLoS ONE. 2006;1:e93 10.1371/journal.pone.0000093 17183725 PMC1762353

[2] Ye X , Hong W , Hao B , et al. PM2.5 promotes human bronchial smooth muscle cell migration via the sonic hedgehog signaling pathway. Respir Res. 2018;19:37 10.1186/s12931-017-0702-y 29499705 PMC5833105

[3] Sun S , Schiller JH , Spinola M , Minna JD . New molecularly targeted therapies for lung cancer. J Clin Invest. 2007;117:2740‐2750. 10.1172/JCI31809 17909619 PMC1994616

[4] Sekido Y , Fong KM , Minna JD . Molecular genetics of lung cancer. Ann Rev Med. 2003;54:73‐87. 10.1146/annurev.med.54.101601.152202 12471176

[5] Watkins DN , Berman DM , Burkholder SG , Wang B , Beachy PA , Baylin SB . Hedgehog signalling within airway epithelial progenitors and in small‐cell lung cancer. Nature. 2003;422:313‐317. 10.1038/nature01493 12629553

[6] Kar S , Deb M , Sengupta D , Shilpi A , Bhutia SK , Patra SK . Intricacies of hedgehog signaling pathways: a perspective in tumorigenesis. Exp Cell Res. 2012;318:1959‐1972. 10.1016/j.yexcr.2012.05.015 22659135

[7] Johnston JJ , Sapp JC , Turner JT , et al. Molecular analysis expands the spectrum of phenotypes associated with GLI3 mutations. Hum Mutat. 2010;31:1142‐1154. 10.1002/humu.21328 20672375 PMC2947617

[8] Ingham PW , Placzek M . Orchestrating ontogenesis: variations on a theme by sonic hedgehog. Nat Rev Genet. 2006;7:841‐850. 10.1038/nrg1969 17047684

[9] Warzecha J , Bonke L , Koehl U , et al. The hedgehog inhibitor cyclopamine induces apoptosis in leukemic cells *in vitro* . Leuk Lymphoma. 2008;49:2383‐2386. 10.1080/10428190802510315 19052992

[10] Bar EE , Chaudhry A , Lin A , et al. Cyclopamine‐mediated hedgehog pathway inhibition depletes stem‐like cancer cells in glioblastoma. Stem Cells. 2007;25:2524‐2533. 10.1634/stemcells.2007-0166 17628016 PMC2610257

[11] Nusslein‐Volhard C , Wieschaus E . Mutations affecting segment number and polarity in Drosophila. Nature. 1980;287:795‐801. 6776413 10.1038/287795a0

[12] Wilson CW , Chuang PT . Mechanism and evolution of cytosolic Hedgehog signal transduction. Development. 2010;137:2079‐2094. 10.1242/dev.045021 20530542 PMC2882129

[13] Kasper M , Regl G , Frischauf AM , Aberger F . GLI transcription factors: mediators of oncogenic Hedgehog signalling. Eur J Cancer. 2006;42:437‐445. 10.1016/j.ejca.2005.08.039 16406505

[14] Zhang Q , Zhang L , Wang B , Ou CY , Chien CT , Jiang J . A hedgehog‐induced BTB protein modulates hedgehog signaling by degrading Ci/Gli transcription factor. Dev Cell. 2006;10:719‐729. 10.1016/j.devcel.2006.05.004 16740475

[15] Goretti E , Wagner DR , Devaux Y . miRNAs as biomarkers of myocardial infarction: a step forward towards personalized medicine? Trends Mol Med. 2014;20:716‐725. 10.1016/j.molmed.2014.10.006 25457620

[16] Zhang JG , Wang JJ , Zhao F , Liu Q , Jiang K , Yang GH . MicroRNA‐21 (miR‐21) represses tumor suppressor PTEN and promotes growth and invasion in non‐small cell lung cancer (NSCLC). Clin Chim Acta. 2010;411:846‐852. 10.1016/j.cca.2010.02.074 20223231

[17] Liu M , Zhang Y , Zhang J , et al. MicroRNA‐1253 suppresses cell proliferation and invasion of non‐small‐cell lung carcinoma by targeting WNT5A. Cell Death Dis. 2018;9:189 10.1038/s41419-017-0218-x 29415994 PMC5833797

[18] Uematsu K , He B , You L , Xu Z , McCormick F , Jablons DM . Activation of the Wnt pathway in non small cell lung cancer: evidence of dishevelled overexpression. Oncogene. 2003;22:7218‐7221. 10.1038/sj.onc.1206817 14562050

[19] Rodriguez‐Blanco J , Schilling NS , Tokhunts R , et al. The hedgehog processing pathway is required for NSCLC growth and survival. Oncogene. 2013;32:2335‐2345. 10.1038/onc.2012.243 22733134 PMC3821972

[20] Zheng X , Dong Q , Zhang X , et al. The coiled‐coil domain of oncogene RASSF 7 inhibits hippo signaling and promotes non‐small cell lung cancer. Oncotarget. 2017;8:78734‐78748. 10.18632/oncotarget.20223 29108261 PMC5667994

[21] Barbieri CE , Baca SC , Lawrence MS , et al. Exome sequencing identifies recurrent SPOP, FOXA1 and MED12 mutations in prostate cancer. Nat Genet. 2012;44:685‐689. 10.1038/ng.2279 22610119 PMC3673022

[22] Liu Y , Melin BS , Rajaraman P , et al. Insight in glioma susceptibility through an analysis of 6p22.3, 12p13.33‐12.1, 17q22‐23.2 and 18q23 SNP genotypes in familial and non‐familial glioma. Hum Genet. 2012;131:1507‐1517. 10.1007/s00439-012-1187-x 22688887 PMC3604903

[23] Keklikoglou I , Koerner C , Schmidt C , et al. MicroRNA‐520/373 family functions as a tumor suppressor in estrogen receptor negative breast cancer by targeting NF‐kappaB and TGF‐beta signaling pathways. Oncogene. 2012;31:4150‐4163. 10.1038/onc.2011.571 22158050

[24] Lu YC , Cheng AJ , Lee LY , et al. MiR‐520b as a novel molecular target for suppressing stemness phenotype of head‐neck cancer by inhibiting CD44. Sci Rep. 2017;7:2042 10.1038/s41598-017-02058-8 28515423 PMC5435724

[25] Jiang J , Hui CC . Hedgehog signaling in development and cancer. Dev Cell. 2008;15:801‐812. 10.1016/j.devcel.2008.11.010 19081070 PMC6443374

[26] Lee RT , Zhao Z , Ingham PW . Hedgehog signalling. Development. 2016;143:367‐372. 10.1242/dev.120154 26839340

[27] Pepicelli CV , Lewis PM , McMahon AP . Sonic hedgehog regulates branching morphogenesis in the mammalian lung. Curr Biol. 1998;8:1083‐1086. 9768363 10.1016/s0960-9822(98)70446-4

[28] Rowitch DH , S‐Jacques B , Lee SM , Flax JD , Snyder EY , McMahon AP . Sonic hedgehog regulates proliferation and inhibits differentiation of CNS precursor cells. The Journal of Neuroscience. 1999;19:8954‐8965. 10516314 10.1523/JNEUROSCI.19-20-08954.1999 PMC6782773

[29] Ingham PW , McMahon AP . Hedgehog signaling in animal development: paradigms and principles. Genes Dev. 2001;15:3059‐3087. 10.1101/gad.938601 11731473

[30] Geng C , Kaochar S , Li M , et al. SPOP regulates prostate epithelial cell proliferation and promotes ubiquitination and turnover of c‐MYC oncoprotein. Oncogene. 2017;36:4767‐4777. 10.1038/onc.2017.80 28414305 PMC5887163

[31] Li G , Ci W , Karmakar S , et al. SPOP promotes tumorigenesis by acting as a key regulatory hub in kidney cancer. Cancer Cell. 2014;25:455‐468. 10.1016/j.ccr.2014.02.007 24656772 PMC4443692

[32] Kwon JE , La M , Oh KH , et al. BTB domain‐containing speckle‐type POZ protein (SPOP) serves as an adaptor of Daxx for ubiquitination by Cul3‐based ubiquitin ligase. J Biol Chem. 2006;281:12664‐12672. 10.1074/jbc.M600204200 16524876

[33] Zhang J , Bu X , Wang H , et al. Cyclin D‐CDK4 kinase destabilizes PD‐L1 via cullin 3‐SPOP to control cancer immune surveillance. Nature. 2018;553:91‐95. 10.1038/nature25015 29160310 PMC5754234

